# Vascular Graft Infections Due to *Listeria* spp.: A Comprehensive Review of the Literature

**DOI:** 10.3390/microorganisms13102262

**Published:** 2025-09-26

**Authors:** Tommaso Lupia, Marco Casarotto, Gianfranco Fonte, Saverio Marena, Francesca Paola Giunta, Fabrizio Buffolo, Silvia Corcione, Francesco Giuseppe De Rosa

**Affiliations:** 1 Department of Medical Sciences, University of Turin, 10126 Turin, Italy; 2 Unit of Infectious Disease, University Hospital Città della Salute e della Scienza di Torino, 10100 Turin, Italy; 3 Internal Medicine Day Hospital, University Hospital Città della Salute e della Scienza di Torino, 10100 Turin, Italy; 4 Division of Nuclear Medicine, University Hospital Città della Salute e della Scienza di Torino, 10100 Turin, Italy; 5 Division of Geographic Medicine, Tufts University School of Medicine, Boston, MA 02111, USA

**Keywords:** *Listeria*, prosthetic infection, vascular graft, listeriosis

## Abstract

Vascular graft infections are rare but serious complications of vascular surgery. *Listeria monocytogenes* represents an exceedingly rare yet potentially life-threatening cause. We present a narrative review according to the Scale for the Assessment of Narrative Review Articles (SANRA) criteria, summarizing the existing literature regarding *Listeria* vascular graft infections. Moreover, we have revised novel potential antibiotics against *Listeria* spp. A systematic search identified 23 studies involving 39 patients. Most infections affected abdominal aortic grafts (50%), with a median interval of 28 months from graft implantation to infection. Common comorbidities included hypertension (30.8%) and diabetes (18%), with 24% of patients being immunocompromised. Diagnosis was supported by blood cultures (45%) and imaging, particularly computed tomography (CT, 90%) and positron emission tomography CT (PET-CT) in selected cases. Surgical intervention was performed in 55% of patients, while 45% were treated medically. Ampicillin, often combined with an aminoglycoside, was the most common first-line therapy. Median treatment duration was 51 days, with some patients requiring lifelong suppressive antibiotic therapy. Mortality was low (one patient), and no recurrences were reported. This review underscores the diagnostic complexity and evolving management strategies in *Listeria* spp. vascular graft infections.

## 1. Introduction

Infection of a vascular graft prosthesis is a rare, although serious, complication of vascular surgery. The overall infection rate is around 2%, varying between 1% and 6% among different types of grafts [1]. Numerous factors appear to contribute, including emergency procedures, demographic characteristics, and various underlying comorbidities [1]. Over the years, causative agents have diversified; current papers indicate that coagulase-negative Staphylococci (CoNS) represent around 42% of cases, followed by *Staphylococcus aureus* at 29% [2]. Other bacteria, such as *Listeria monocytogenes*, are rare. *Listeria* spp. is a facultative anaerobic, Gram-positive bacillus that proliferates on blood agar and exhibits partial β-hemolysis [2]. There are seven recognized species of *Listeria*, although *L. monocytogenes* is the predominant source of human illnesses [2,3]. It often impacts pregnant women, infants, older adults, and the immunocompromised population [2,3]. This vulnerability can be somewhat elucidated by the dual nature of the response to this infection, which encompasses both innate and adaptive immunity, heavily dependent on macrophage activation through T-cell lymphokines [4]. *L. monocytogenes* is prevalent in nature, particularly among the fecal flora of animals, and is a significant contributor to zoonotic diseases, with a case-fatality rate that may attain 20% [2]. Listeriosis, excluding perinatal instances, is predominantly foodborne. Numerous foods have been recognized as vectors of illness, including milk, soft cheese, ready-to-eat pork products, hot dogs, and delicatessen turkey, among others [5,6,7]. Other uncommon mechanisms of transmission have been documented, including maternal–fetal transmission through the placenta, exposure during parturition, or direct contact with aborted calves and infected poultry, which is mainly reported among agricultural workers and veterinarians [2,3]. In susceptible people, listeriosis may manifest in three different clinical presentations: bacteremia (with or without septic shock); central nervous system involvement, commonly meningitis or meningoencephalitis; or pregnancy-associated and neonatal listeriosis; which account, respectively, for 31%, 52%, and 14% of the cases reported in the literature [2]. Moreover, in otherwise healthy persons, it often results in self-limiting febrile gastroenteritis [1,2]. Grif and colleagues have recorded the temporary presence of *L. monocytogenes* in the feces of healthy persons [8].

Uncommon infections have been reported affecting the skin (e.g., pyogranulomatous rash), peritoneal cavity, arthroskeletal tissue, lungs and pleural cavity, cardiovascular system, urinary tract, biliary tract, and the eye, each generally representing fewer than 1% of the overall listeriosis cases [2,9,10,11,12]. In 2010, the global burden of the illness comprised 23,150 cases, 5463 fatalities, and 172,823 disability-adjusted life years [2].

Few studies have documented vascular prosthesis infections caused by *L. monocytogenes* [13,14,15], and even fewer studies have addressed the involvement of endovascular prostheses. This paper aims to present a comprehensive review of the literature of vascular prosthesis infection caused by *L. monocytogenes*. Moreover, we have revised the literature regarding novel antibiotics available against this bacterium.

## 2. Methods

**Figure 1:**
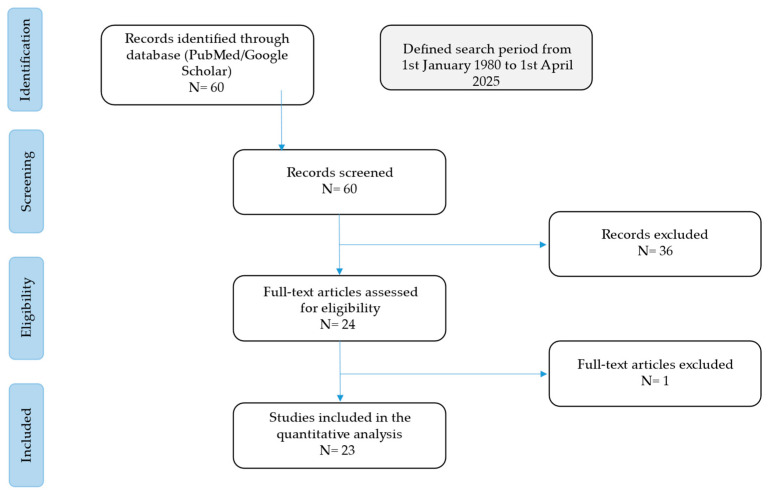
Flow-chart of the studies revised in the narrative review.

The current narrative review followed the Scale for the Assessment of Narrative Review Articles (SANRA) flow-chart (Figure 1) [16].

The main aim of this work is to summarize current evidence on vascular graft infections due to *Listeria* spp., focusing on clinical presentations, management, and outcomes.

A search was run on Google Scholar and PubMed using the terms (“*Listeria monocytogenes*” OR “Listeriosis” OR “Listeria”) AND (“Aortic Diseases” OR “Aortic Aneurysm” OR “Aortic Graft Infection” OR “Vascular Graft Infection” OR “Graft Infection” OR “Prosthetic Infection” OR “Endograft Infection”). Results were limited to papers published between 1 January 1980 and 1 April 2025. Studies were filtered for practice guidelines, guidelines, meta-analyses, systematic reviews, narrative reviews, case series, and case reports. The results were filtered to include only humans, and patients over 18 years old.

Our search strategy permitted the identification of 60 papers, of which 36 were excluded by title and abstract evaluation. Then, the reviewers studied titles and abstracts. Subsequently, 24 papers were included. One paper was excluded because it was not possible to find the full text. Finally, a quality assessment of full-text studies was performed by two independent reviewers (MC and TL). The researchers reviewed the summary of all articles and ultimately used data from full articles to compile this review paper. The researchers assessed the inclusion of all titles and abstracts without language limitations in English. We duplicated other studies previously included and excluded papers with no methods described, along with papers not strictly related to the aim of the study.

Moreover, we have revised the literature regarding new antibiotic molecules with an activity in vitro or in vivo against *Listeria* spp.

We performed descriptive statistics on the entire study population. Data were analyzed using standard statistical methods. Variables were described with medians, absolute values, and rates. Three studies were discussed separately from the others because they included both endovascular graft and non-graft infections.

## 3. Results

The final analysis comprised 23 papers with a total of 39 participants. We identified 18 case reports and two case series, encompassing a total of 20 patients, as presented in Table 1. Additionally, three publications were retrospective studies involving 19 patients, and their findings are elaborated upon in a specific paragraph.

**Table 1:** Clinical characteristics.

Author	Year	Type of Study	Patients	Sex	Age	Type of Graft	Other Seedings		Time from Prosthesis Implantation
Rohde et al. [17]	2004	Case Report	1	M	51	Ascendens Aortic prosthesis	No		6 months
Miranda et al. [18]	2022	Case Report	1	M	72	Abdominal Aortic prosthesis	No		9 years
Ahadzada et al. [19]	2021	Case Report	1	M	80	Abdominal Aortic prosthesis	No		7 months
Berchiolli et al. [20]	2022	Case Report	1	M	72	Abdominal Aortic prosthesis	No		3 years
Lauk et al. [21]	2020	Case Report	1	M	70	Thoracic and abdominal Aortic prosthesis	No		5 years
Chavada et al. [22]	2014	Case Series	1	M	83	Femoro-popliteal graft	No		2 years
Ma et al. [23]	2019	Case Report	1	M	66	Abdominal Aortic prosthesis	Psoas abscess		18 months
Tanner-Steinmann et al. [24]	2011	Case Report	1	M	59	Abdominal Aortic prosthesis	No		2 years
Saleem et al. [25]	2008	Case Report	1	M	67	Abdominal Aortic prosthesis	No		10 months
Foulex et al. [26]	2019	Case Report	1	M	76	Abdominal Aortic prosthesis	No		NA
Heikkinen et al. [27]	1999	Case Report	1	M	77	Abdominal Aortic prosthesis	No		3 months
Silvestri et al. [28]	2017	Case Report	1	M	72	Thoracic and abdominal Aortic prosthesis	No		7 years
Mercurio et al. [29]	2021	Case Report	1	M	83	Abdominal Aortic prosthesis	Spondylodiscitis		1 months
Zardi et al. [30]	2022	Case Report	1	M	71	Aorto-bi-iliac endograft	No		2 months
Gauto et al. [31]	1992	Case Series	1	M	59	Femoro-popliteal graft	Endocarditis		1 year
De Nietet al. [32]	2018	Case report	1	M	81	Abdominal Aortic prosthesis	No		5 years
Zeitlin et al. [33]	1982	Case report	1	F	51	Arteriovenous graft radial artery antecubital vein	No		5 months
Van Noyen et al. [34]	1993	Case report	1	M	75	Femoro-popliteal bypass	No		3 years
Heysell et al. [35]	2015	Case report	1	F	68	Thoracic Aortic prosthesis	No		2 years
Walker et al. [36]	2015	Case report	1	M	70	Femoro-popliteal graft	No		1 year
					
**Author**	**Comorbidities**		**Risk Factors**	***Listeria* spp. Previous Infection**			**Blood Culture**	**Bio Mol**	**Antimicrobial Susceptibility**
Rohde et al. [17]	Arterial hypertension, hyperlipoproteinemia		NA	Yes (bacteriemia 7 weeks before admission)			*Listeria monocytogenes*	No	S penicillin, gentamicin, trimethoprim-sulfamethoxazole
Miranda et al. [18]	NA		NA	No			Negative	No	Not reported
Ahadzada et al. [19]	Arterial hypertension, hyperlipoproteinemia, CAD		NA	Not confirmed (non-bloody diarrhea 4 weeks before admission)			Negative	No	Not reported
Berchiolli et al. [20]	Arterial hypertension, DM, CAD		NA	No			*Listeria monocytogenes*	No	Not reported
Lauk et al. [21]	CAD		NA	No			Negative	Yes	Not reported
Chavada et al. [22]	Atrial fibrillation		Undercooked meat	No			Not performed	No	S penicillin, gentamicin, trimethoprim-sulfamethoxazole
Ma et al. [23]	Arterial hypertension		NA	Not confirmed (abdominal pain and fever 4 weeks before admission)			*Listeria monocytogenes*	No	S penicillin, gentamicin, trimethoprim-sulfamethoxazole
Tanner-Steinmann et al. [24]	Kidney transplant		NA	Not confirmed (non-bloody diarrhea 8 weeks before admission)			Negative	No	Not reported
Saleem et al. [25]	COPD, DM, CAD		Livestock farmer	No			Negative	No	Not reported
Foulex et al. [26]	COPD, DM, CAD		NA	Not confirmed (non-bloody diarrhea 10 days before admission)			Negative	Yes	S penicillin, gentamicin, trimethoprim-sulfamethoxazole
Heikkinen et al. [27]	Obesity, arterial hypertension		NA	No			Negative	No	S penicillin, gentamicin, trimethoprim-sulfamethoxazole
Silvestri et al. [28]	Giant cell arteritis, DM, arterial hypertension		NA	Not confirmed (abdominal pain and fever 8 weeks before admission)			*Listeria monocytogenes*	No	Not reported
Mercurio et al. [29]	DM, arterial hypertension, PM		NA	No			*Listeria monocytogenes*	No	S penicillin, gentamicin, trimethoprim-sulfamethoxazole
Zardi et al. [30]	DM, arterial hypertension, splenectomy		NA	No			Negative	No	Not reported
Gauto et al. [31]	Rheumatoid arthritis, CAD, arterial hypertension		NA	No			*Listeria monocytogenes*	No	Not reported
De Nietet al. [32]	Arterial hypertension		NA	No			Negative	No	S penicillin, gentamicin, trimethoprim-sulfamethoxazole
Zeitlin et al. [33]	DM, arterial hypertension, CKD		NA	No			*Listeria monocytogenes*	No	S penicillin, gentamicin, trimethoprim-sulfamethoxazole
Van Noyen et al. [34]	Arterial hypertension, COPD		NA	No			Not performed	No	S penicillin, gentamicin, trimethoprim-sulfamethoxazole
Heysell et al. [35]	Idiopathic cardiomyopathy, AF		Unpasteurized dairy	Not confirmed (diarrhea 3 months before admission)			*Listeria monocytogenes*	No	S penicillin, gentamicin, trimethoprim-sulfamethoxazole
Walker et al. [36]	NA		NA	No			*Listeria monocytogenes*	Yes	Not reported
						
**Author**	**Stool Culture**		**Graft Culture**	**CT**			**MRI**	**PET/TC**	**Follow-Up Radiologico**
Rohde et al. [17]	No		No	Yes			No	Yes	No
Miranda et al. [18]	No		Positive	Yes			No	No	No
Ahadzada et al. [19]	No		No	Yes			No	Yes	No
Berchiolli et al. [20]	No		Positive	Yes			No	Yes	No
Lauk et al. [21]	No		No	Yes			No	No	No
Chavada et al. [22]	No		Positive	Yes			No	No	No
Ma et al. [23]	Negative		No	Yes			No	No	Yes
Tanner-Steinmann et al. [24]	No		No	Yes			Yes	No	Yes
Saleem et al. [25]	No		No	Yes			No	Yes	Yes
Foulex et al. [26]	Negative		Positive	Yes			No	Yes	Yes
Heikkinen et al. [27]	No		Positive	Yes			No	No	Yes
Silvestri et al. [28]	NA		NA	NA			NA	NA	NA
Mercurio et al. [29]	No		No	Yes			Yes	Yes	No
Zardi et al. [30]	No		Positive	Yes			No	No	No
Gauto et al. [31]	No		No	Yes			No	No	No
De Nietet al. [32]	No		No	Yes			No	Yes	No
Zeitlin et al. [33]	No		No	No			No	No	No
Van Noyen et al. [34]	No		Positive	Yes			No	No	Yes
Heysell et al. [35]	No		No	Yes			No	No	Yes
Walker et al. [36]	No		Negative	Yes			No	No	Yes

Abbreviations: M: male; F: female; NA: not available; DM: diabetes mellitus; CKD: chronic kidney disease; CAD: coronary artery disease; PM: pacemaker; S: susceptible; COPD: chronic obstructive pulmonary disease, AF: arterial fibrillation.

**Figure 2:**
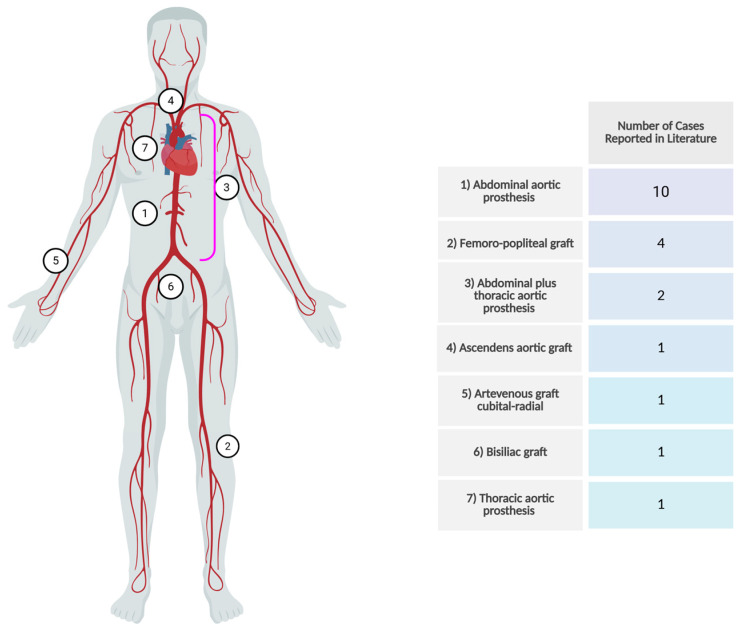
Vascular distribution of *Listeria* spp. infections according to revised literature and findings reported in “type of graft” column in Table 1.

The median age was 70 years (range 51–83), with a predominance of males (N = 18; 90%). Two studies did not report patient comorbidities; nevertheless, among the others, the most prevalent condition was arterial hypertension (N = 12; 30.8%), followed by diabetes mellitus (DM) in seven instances (18%), whereas coronary artery disease was noted in five individuals (25%). Three individuals (15%) had a diagnosis of COPD in their medical history. Notably, four patients (24%) were immunocompromised for various reasons: one was a kidney transplant recipient, one was on steroid treatment for giant cell arteritis, one was on drugs for rheumatoid arthritis, and one had undergone a splenectomy some years prior to the infection. The majority of infections were associated with abdominal aortic grafts (N = 10; 50%), followed by femoro-popliteal grafts (N = 4; 20%) and thoracic aortic grafts (N = 2; 10%). Other types of less common vascular prostheses involvement are depicted in Figure 2.

The duration from implant to graft infection exhibited considerable variability, with a median of 28 months (range 1 month to 9 years). Only three patients (15%) reported traditional risk factors for listeriosis, such as the consumption of undercooked meat, employment as a livestock farmer, and the intake of unpasteurized dairy products. Only one patient had confirmed bacteremia caused by *L. monocytogenes* seven weeks prior to the detection of transplant infection. Six patients (30%) had symptoms (i.e., non-bloody diarrhea, fever, or stomach discomfort) indicative of *Listeria* spp. infection in the three months before admission. Nine (45%) had positive blood cultures, consistently for *L. monocytogenes*. In seven cases, graft cultures validated the diagnosis by yielding growth of *L. monocytogenes*. A CT scan with contrast was conducted in 18 instances (90%), whilst MRI was utilized for certain occurrences. A PET scan was conducted in seven instances (35%).

Three instances of infection seeding were documented, specifically, one case of endocarditis, one case of psoas abscess, and one case of spondylodiscitis. Antimicrobial susceptibility was reported in 10 individuals (50%), with all isolates demonstrating susceptibility to penicillin, aminoglycosides, and trimethoprim-sulfamethoxazole.

In terms of treatment, 11 patients (55%) had surgical intervention and source control, whereas 9 patients (45%) were managed solely by medical therapy. ßeta-lactam constituted the most recommended antibiotic class for first-line therapy, with IV ampicillin administered in eight instances (40%), followed by IV amoxicillin or amoxicillin-clavulanate (N = 4; 20%), as reported in Table 2.

**Table 2:** Treatments and outcomes of patients collected from the literature.

Author	Percutaneous Drainage	Surgery	Treatment	Duration	SAT (Lifelong) or Consolidation (>6 Months)	Recurrence	Outcome
Rohde et al. [17]	No	No	ampicillin (6 weeks) + gentamicin (4 weeks) followed by amoxicillin (8 weeks)	109 days	No	No	12 months FU
Miranda et al. [18]	No	Yes	ampicillin + gentamicin	28 days	SAT amoxicillin	No	6 months FU
Ahadzada et al. [19]	Yes	No	IV benzylpenicillin	48 days	SAT amoxicillin	No	10 months FU
Berchiolli et al. [20]	Yes	Yes	ampicillin-sulbactam	Not reported	Not reported	No	3 months FU
Lauk et al. [21]	Yes	No	meropenem + vancomycin (1 week) followed by amoxicillin + gentamicin (21 days)	29 days	SAT piperacillin-tazobactam	No	24 months FU
Chavada et al. [22]	No	Yes	ampicillin (1 week) followed by meropenem (5 weeks)	42 days	Consolidation amoxicillin 6 months	No	6 months FU
Ma et al. [23]	No	No	ampicillin (4 weeks) + gentamicin for 8 weeks followed by trimetoprim-sulfametoxazole IV for 12 days	42 days	Consolidation trimetoprim-sulfametoxazole 13.5 months	No	20 months FU
Tanner-Steinmann et al. [24]	Yes	Yes	ampicillin (4 weeks) followed by ampicillin + gentamicin (4 week)	42 days	Consolidation trimetoprim-sulfametoxazole 24 months	No	30 months FU
Saleem et al. [25]	Yes	No	amoxicillin-clavulanic + trimetoprim-sulfametoxazole	28 days	Consolidation trimetoprim-sulfametoxazole 6 months	No	12 months FU
Foulex et al. [26]	No	Yes	amoxicillin	42 days	No	No	6 months FU
Heikkinen et al. [27]	Yes	Yes	ampicillin + netilmicin (2 weeks) followed by doxycycline (4 weeks)	42 days	No	No	10 months FU
Silvestri et al. [28]	NA	NA	NA	NA	Not described	No	NA
Mercurio et al. [29]	Yes	No	ampicillin + gentamicin (2 weeks) followed by ampicillin (4 weeks)	42 days	No	No	12 months FU
Zardi et al. [30]	No	Yes	meropenem + vancomycin (3 weeks) followed byclaritromicine (12 weeks)	105 days	No	No	9 months FU
Gauto et al. [31]	No	No	ampicillin + trimetoprim-sulfametoxazole	56 days	No	No	18 months FU
De Niet et al. [32]	Yes	Yes	amoxicillin	28 days	SAT trimetoprim-sulfametoxazole	No	6 months FU
Zeitlin et al. [33]	No	Yes	vancomycin	42 days	No	No	9 months FU
Van Noyen et al. [34]	No	Yes	amoxicillin-clavulanic acid + gentamicin	NA	Not described		Death
Heysell et al. [35]	No	No	vancomycin	42 days	SAT doxicycline	No	36 months FU
Walker et al. [36]	No	Yes	Not described	NA	Not described	No	Not reported

Abbreviations: SAT: suppressive antibiotic treatment; FU: follow-up.

Combination treatment was administered in 10 instances (50%), with IV gentamicin being the most used adjunct antibiotic (N = 6; 30%). The median duration of first-line treatment was 51 days (ranging from 28 to 109 days). Four patients (20%) had consolidation antibiotic therapy, characterized by a minimum duration of 6 months of treatment. Lifelong suppressive antibiotic therapy was prescribed in five patients (25%). Trimethoprim-sulfamethoxazole was the most prescribed antibiotic for consolidation therapy (N = 3, 75%), but amoxicillin was the chosen antibiotic for maintenance therapy (N = 2, 40%).

The median follow-up time was 12 months (ranging from 3 to 36 months). One patient died during therapy. The follow-up period was not recorded in two instances. No incidences of recurrence were documented.

### 3.1. Retrospective Studies

Despite the absence of specific clinical data, we chose to incorporate three retrospective investigations due to the substantial number of cases documented about patients with *L. monocytogenes* vascular graft infections.

Ljungquist and colleagues performed retrospective observational single-center research in Sweden to elucidate the technique of direct aneurysm sac-guided suction to identify the microbiological etiology in a cohort of patients with vascular graft or endograft infections. Four *L. monocytogenes* graft infections were identified, leading to the conclusion that this surgical method may effectively ascertain the microbiological etiology of both open and endovascular graft infections, especially when conventional culture techniques are integrated with 16S rRNA/18S rRNA [37].

Cernohorsky and colleagues presented a retrospective cohort study conducted in two big hospitals in the Netherlands to elucidate the occurrence of endograft infection following endovascular repair of abdominal (EVAR) and thoracic aortic aneurysm (TEVAR), identifying one case attributed to *L. monocytogenes*. The patient received conservative surgical intervention and was treated with a combined therapy of intravenous amoxicillin/clavulanic acid and intravenous trimethoprim-sulfamethoxazole, followed by consolidation therapy with oral trimethoprim-sulfamethoxazole [38].

Shoai-Tehrani and colleagues retrospectively characterized 71 culture-confirmed *Listeria monocytogenes*-associated endovascular infections reported to the French National Reference Centre for *Listeria* from 1993 to 2018. Specifically, 14 instances pertained to prosthetic endovascular infections. The stated median age was 74, with a predominant majority of men (91%). The predominant location of infection was the descending aorta (N = 7; 50%), followed by the femoro-popliteal graft (N = 6; 42%). Cardiovascular disease was the predominant comorbidity reported (76%), encompassing hypertension (27%), coronary artery disease (23%), and atrial fibrillation (13%). Immunosuppressive diseases were identified in 50% of the individuals [39].

All patients were administered at least one antibiotic active against *Listeria* spp., with ßeta-lactams used in 90% of instances. Among all patients, 84% were provided with combination therapy.

Most of those patients started combination antimicrobial treatment with amoxicillin and an aminoglycoside [40]. Shoai-Tehrani and associates discovered that the death rate was markedly elevated in individuals with endocarditis compared to those with vascular infections (41% versus 12%, *p* = 0.009).

### 3.2. Potential Novel Antibiotics Against Listeria spp.

Given the need for prolonged therapies in this cohort of patients, we aimed to explore the available literature concerning novel compounds active against *Listeria* monocytogenes. Oritavancin, a novel long-acting lipoglycopeptide, demonstrates promising in vitro activity against *Listeria monocytogenes* isolates, as assessed by broth microdilution, with MIC 50 findings of ≤0.008 mg/L, as reported in Table 3 [41].

Additionally, Dalbavancin was assessed in seldom isolated Gram-positive bacteria, demonstrating promising in vitro efficacy against *Listeria monocytogenes* (MIC 0.06/0.12 μg/mL) isolates [42]. Sader et al. found restricted efficacy of the fifth-generation cephalosporin, ceftaroline, against some strains of *Listeria monocytogenes* [43]. Daptomycin was assessed against clinical isolates of infrequently reported Gram-positive microorganisms, including viridans group streptococci, *Corynebacterium* spp., *Micrococcus* spp., and *L. monocytogenes*, which demonstrated elevated daptomycin minimum inhibitory concentrations (MIC 50 and MIC 90, 2 and 4 μg/mL, respectively) compared to other evaluated organisms [44]. Additionally, a promising intracellular effectiveness of tedizolid against *L. monocytogenes* (MIC 0.125 mg/L) isolates was documented in a pioneering work [45,46]. Furthermore, the inherent properties particular to the antibiotic class suggested a possible efficacy of meropenem/vaborbactam, delafloxacin, plazomicin, tigecycline, and eravacycline against *L. monocytogenes*, despite the absence of in vitro or in vivo evidence for these innovative therapeutic drugs at the time of this research. To our knowledge, no ongoing trials involving novel antibiotics and *L. monocytogenes* are registered at the time of our paper submission.

## 4. Discussion

In this paper we aimed to review the literature on vascular graft infections due to *Listeria monocytogenes*, with a focus on treatment and the activity of novel antibiotics against these bacteria.

Most of the patients (90%) included in this review were males, with a median age of 74 years old. According to recent evidence, male sex and older adults are at higher risk for vascular graft infections compared to the general population, and older males seem to have a higher rate of vascular surgery interventions compared to the general population [47]. Moreover, *Listeria* spp. infection is most often seen in the first 30 days of life or in patients older than 60 [48], and a recent meta-analysis showed an increase in listeriosis-related mortality with age [2]. This age-related higher prevalence in the elderly was probably linked to immunosenescence, impaired innate and adaptive immunity, and higher prevalence of immunocompromised comorbidities in older people [2,3,4]. Cardiovascular diseases, including arterial hypertension, were the most frequently reported comorbidities in the general population, probably reflecting the prevalence of these conditions in the population collected in this review [49]. The second most frequently reported comorbidity was diabetes mellitus (18%). This finding is consistent with data from the literature, such as those from the MONNALISA registry, in which 22% of patients with invasive listeriosis were affected by diabetes [50]. Although the patients were, on average, older, it is possible that impaired immunity in individuals with diabetes contributes to the increased risk of invasive *Listeria monocytogenes* infections. Excluding diabetes, only a minor part of patients reported other conditions causing immunodepression (25%) such rheumatological diseases and transplant recipients, as reported in Table 1.

Abdominal aortic grafts were the most common site of vascular infections in the papers included in this review; this probably reflects the epidemiology of surgery intervention [50]. Time from vascular prosthesis implantation to infection was highly variable, probably reflecting different times of exposure. Regarding *Listeria* spp. exposure, only a minority of the studies included in the review provided information on exposures presenting a risk of this infection. In fact, *Listeria monocytogenes* can theoretically contaminate a large and diverse array of food products, so it can be difficult to spot the source of infection [2]. We found that the mean time from possible *Listeria* spp. exposure or symptoms compatible with gastroenteric *Listeria* infection to the vascular graft infection was about 6 weeks. This is in line with the infection pathophysiology reported in the literature from experimental data in animals and clinical information. Initially, *Listeria monocytogenes* traverses the intestinal barrier and translocates to mesenteric lymph nodes and the liver and spleen [2]. Early stages are usually asymptomatic but can sometimes manifest as nonspecific febrile illness and gastroenteritis [2].

The most common microbiological diagnosis was through positive blood cultures; nevertheless, more than half of the patients (55%) had negative blood cultures, and microbiologic diagnosis was obtained via culturing percutaneous drained aspirations or biopsy taken during surgery.

Although an injected CT scan was the most common radiological diagnostic method in this literature review, we found that PET/TC was used during the diagnostic process in seven cases (35%), and for follow-up in three cases (15%). As recently suggested by the Management of Aortic Graft Infection Collaboration (MAGIC) [51], the diagnosis of vascular infection derives from the combination of clinical, biochemical, and microbiological findings, as well as imaging.

PET/TC reported high sensitivity and negative predictive value in the diagnosis of vascular graft infections and a potential role in imaging follow-up during treatment [51,52]. However, given its lower specificity, positive results should be confirmed, for example, with white blood cell scintigraphy, especially in early graft infections [52]. No definitive data on white blood cell scintigraphy were available from the article collected.

Most of the patients were treated conservatively with a combination of ampicillin plus an aminoglycoside. Nonetheless, it must be noted that clear evidence supporting the efficacy of combination therapy in *Listeria monocytogenes* infections is lacking in the literature [53]. Almost half of patients (45%) were treated with medical treatment (without source control being feasible) and 55% were treated with both surgery and antibiotics.

Only one patient died, and no case of relapse or recrudescence were reported. This result, although limited by the low sample size of this cohort, diverges from other results in the literature, where mortality for bacteriemia is around 27%.

Regarding the duration of antibiotic treatment, we found considerable heterogeneity in the literature. Most frequently, at least 4 to 6 weeks of first line treatment were prescribed, followed by either consolidation therapy (defined as at least 6 months of treatment), lifelong suppressive antibiotic therapy in similar proportions (25% vs. 20%, respectively), or nothing (35%).

In the review of novel compounds with potential in vitro or in vivo activity against *Listeria* spp. we have reported interesting preliminary data on the in vitro activity of two new long-acting antibiotics, dalbavancin and oritavancin [41,42,54,55]; nevertheless, data should be confirmed in vivo and in larger studies. Given the need for prolonged therapies in this cohort of patients, the long-acting compounds profile in terms of efficacy and safety seems promising, also due to the potential anti-biofilm activity in graft infections [54,55]. Regarding antibiotics with a potential activity against *Listeria* spp., we also look with interest to tedizolid and delafloxacin, especially for the possibility of both oral and intravenous administration and the interesting pharmacokinetic profile and vascular graft penetration [56,57]. Despite that, data regarding delafloxacin and tedizolid activity were only based on in vitro activity and data should be confirmed in vivo and in larger studies.

## 5. Conclusions

In conclusion, while vascular graft infections due to *Listeria monocytogenes* remain a rare clinical entity, our review highlights the need for greater standardization in diagnostic and therapeutic approaches. The current literature, based largely on retrospective data and case reports, underscores the importance of heightened clinical suspicion. At risk patients could be older male adults with abdominal aortic grafts, including those without a classical immunocompromised past medical history. PET-CT may be considered in the diagnosis and follow-up of vascular graft infections without surgical indications, especially in case of prolonged or suppressive antibiotic treatment. Further research is needed to better define optimal management strategies for vascular graft infections due to *Listeria monocytogenes*, including duration of antibiotic therapy, as well as the need for surgical intervention.

## Figures and Tables

**Table 3:** Novel antibiotics against *Listeria monocytogenes*.

Lipoglycopeptides	
Oritavancin	Activity in vitro
Dalbavancin	Activity in vitro
Daptomycin	Activity in combination therapy
**Oxazolidinones**	
Tedizolid	Activity in vitro
**Carbapenems**	
Meropenem/vaborbactam	Potential activity based on antibiotic class
**Cephalosporins**	
Ceftaroline	Activity in vitro
**Aminoglycosides**	
Plazomicin	Potential activity based on antibiotic class
**Fluoroquinolones**	
Delafloxacin	Potential activity based on antibiotic class
**Tetracyclines**	
Eravacycline	Potential activity based on antibiotic class
Tigecycline	Potential activity based on antibiotic class

## Data Availability

No new data were created or analyzed in this study. Data sharing is not applicable to this article.

## References

[1] Chiesa R., Astore D., Frigerio S., Garriboli L., Piccolo G., Castellano R., Scalamogna M., Odero A., Pirrelli S., Biasi G. (2002). Vascular prosthetic graft infection: Epidemiology, bacteriology, pathogenesis and treatment. Acta Chir. Belg..

[2] Koopmans M.M., Brouwer M.C., Vázquez-Boland J.A., van de Beek D. (2023). Human Listeriosis. Clin. Microbiol. Rev..

[3] Swaminathan B., Gerner-Smidt P. (2007). The epidemiology of human listeriosis. Microbes Infect..

[4] Zenewicz L.A., Shen H. (2007). Innate and adaptive immune responses to *Listeria monocytogenes*: A short overview. Microbes Infect..

[5] Farber J.M., Peterkin P.I., Carter A.O., Varughese P.V., Ashton F.E., Ewan E.P. (1991). Neonatal listeriosis due to cross-infection confirmed by isoenzyme typing and DNA fingerprinting. J. Infect. Dis..

[6] Schwartz B., Hexter D., Broome C.V., Hightower A.W., Hirschhorn R.B., Porter J.D., Hayes P.S., Bibb W.F., Lorber B., Faris D.G. (1989). Investigation of an outbreak of listeriosis: New hypotheses for the etiology of epidemic *Listeria monocytogenes* infections. J. Infect. Dis..

[7] Riedo F.X., Pinner R.W., Tosca M.L., Cartter M.L., Graves L.M., Reeves M.W., Weaver R.E., Plikaytis B.D., Broome C.V. (1994). A point-source foodborne listeriosis outbreak: Documented incubation period and possible mild illness. J. Infect. Dis..

[8] Grif K., Patscheider G., Dierich M.P., Allerberger F. (2003). Incidence of fecal carriage of Listeria monocytogenes in three healthy volunteers: A one-year prospective stool survey. Eur. J. Clin. Microbiol. Infect. Dis..

[9] Antolín J., Gutierrez A., Segoviano R., López R., Ciguenza R. (2008). Endocarditis due to Listeria: Description of two cases and review of the literature. Eur. J. Intern. Med..

[10] Pilmis B., Leclercq A., Maury M.M., Moura A., Bracq-Dieye H., Thouvenot P., Valès G., Lecuit M., Charlier C., Cutaneous Listeriosis Study Group (2020). Cutaneous listeriosis, a case series of 16 consecutive patients over 25 years. J. Infect..

[11] Danion F., Maury M.M., Leclercq A., Moura A., Perronne V., Léotard S., Dary M., Tanguy B., Bracq-Dieye H., Thouvenot P. (2017). Listeria monocytogenes isolation from urine: A series of 15 cases and review. Clin. Microbiol. Infect..

[12] Charlier C., Leclercq A., Cazenave B., Desplaces N., Travier L., Cantinelli T., Lortholary O., Goulet V., Le Monnier A., Lecuit M. (2012). Listeria monocytogenes-associated joint and bone infections: A study of 43 consecutive cases. Clin. Infect. Dis..

[13] Murphy K., Al-Jundi W., Nawaz S. (2013). Mycotic aneurysms of the abdominal aorta due to Listeria monocytogenes. Int. J. Surg. Case Rep..

[14] Oderich G.S., Panneton J.M., Bower T.C., Cherry K.J., Rowland C.M., Noel A.A., Hallett J.W., Gloviczki P. (2001). Infected aortic aneurysms: Aggressive presentation, complicated early outcome, but durable results. J. Vasc. Surg..

[15] Brossier J., Lesprit P., Marzelle J., Allaire E., Becquemin J.P., Desgranges P. (2010). New bacteriological patterns in primary infected aorto-iliac aneurysms: A single-centre experience. Eur. J. Vasc. Endovasc. Surg..

[16] Baethge C., Goldbeck-Wood S., Mertens S. (2019). SANRA-a scale for the quality assessment of narrative review articles. Res. Integr. Peer Rev..

[17] Rohde H., Horstkotte M.A., Loeper S., Aberle J., Jenicke L., Lampidis R., Mack D. (2004). Recurrent Listeria monocytogenes aortic graft infection: Confirmation of relapse by molecular subtyping. Diagn. Microbiol. Infect. Dis..

[18] Miranda J.A., Khouqeer A., Livesay J.J., Montero-Baker M. (2022). Very Late Aortic Endograft Infection with Listeria monocytogenes in an Elderly Man. Tex. Heart Inst. J..

[19] Ahadzada Z., Ghaly P., Farmer E., Ahmad M. (2022). Listeria monocytogenes endograft infection after fenestrated endovascular aneurysm repair-a case report. J. Vasc. Surg. Cases Innov. Tech..

[20] Berchiolli R., Bertagna G., Erba P.A., Ferrari M., Troisi N. (2022). Listeria abdominal endograft infection miming pseudoaneurysm treated with in-situ aortic reconstruction: A case report. Eur. Rev. Med. Pharmacol. Sci..

[21] Lauk O., Fulchini R., Hasse B.K., Schmitt-Opitz I. (2020). Aortobronchial fistula and Listeria endograft infection after repeated T/EVAR: A rare combination. BMJ Case Rep..

[22] Chavada R., Keighley C., Quadri S., Asghari R., Hofmeyr A., Foo H. (2014). Uncommon manifestations of Listeria monocytogenes infection. BMC Infect. Dis..

[23] Ma J.W., Hu S.Y., Lin T.C., Tsai C.A. (2019). Psoas abscess associated with aortic endograft infection caused by bacteremia of Listeria monocytogenes: A case report and literature review (CARE Complaint). Medicine.

[24] Tanner-Steinmann B., Boggian K. (2011). Vascular Endograft Infection with Listeria monocytogenes reated with Surgical Debridement but without Graft Removal. Case Rep. Med..

[25] Saleem B.R., Berger P., Zeebregts C.J., Slart R.H., Verhoeven E.L., van den Dungen J.J. (2008). Periaortic endograft infection due to Listeria monocytogenes treated with graft preservation. J. Vasc. Surg..

[26] Foulex A., Coen M., Cherkaoui A., Lazarevic V., Gaïa N., Leo S., Girard M., Mugnai D., Schrenzel J. (2019). Listeria monocytogenes infectious periaortitis: A case report from the infectious disease standpoint. BMC Infect. Dis..

[27] Heikkinen L., Valtonen M., Lepäntalo M., Saimanen E., Järvinen A. (1999). Infrarenal endoluminal bifurcated stent graft infected with Listeria monocytogenes. J. Vasc. Surg..

[28] Silvestri V., Isernia G. (2017). Suspected Giant Cell Aortitis: From Multiple Aortic Structural Damage to Fatal Listeria Sepsis, a Case Report. Ann. Vasc. Surg..

[29] Mercurio M., Sanzo V., Rava A., Galasso O., Gasparini G. (2021). Spondylodiscitis After Endovascular Aortic Repair Due to Noninvasive Listeriosis: A Case Report. JBJS Case Connect..

[30] Zardi E.M., Montelione N., Catanese V., Gabellini T., Caricato M., Zardi D.M., Spinelli F., Stilo F. (2022). First case of aorto-bi-iliac endograft thrombotic infection by Listeria monocytogenes: A case report. Exp. Ther. Med..

[31] Gauto A.R., Cone L.A., Woodard D.R., Mahler R.J., Lynch R.D., Stoltzman D.H. (1992). Arterial infections due to Listeria monocytogenes: Report of four cases and review of world literature. Clin. Infect. Dis..

[32] de Niet A., van Schaik P.M., Saleem B.R., Zeebregts C.J., Tielliu I.F.J. (2018). Endovascular Aneurysm Repair Complicated with Type Ia Endoleak and Presumable Infection Treated with a Fenestrated Endograft. Aorta.

[33] Zeitlin J., Carvounis C.P., Murphy R.G., Tortora G.T. (1982). Graft infection and bacteremia with Listeria monocytogenes in a patient receiving hemodialysis. Arch. Intern. Med..

[34] Van Noyen R., Reybrouck R., Peeters P., Verheyen L., Vandepitte J. (1993). Listeria monocytogenes infection of a prosthetic vascular graft. Infection.

[35] Heysell S.K., Hughes M.A. (2016). Listeria monocytogenes Endovascular Graft Infection. Open Forum Infect. Dis..

[36] Walker A.S., Smith J.P., Andersen C.A., Daab L.J. (2015). Listeria monocytogenes infection of a popliteal artery stent graft. Case Stud. Surg..

[37] Ljungquist O., Dias N., Haidl S., Sonesson B., Sörelius K., Ahl J. (2021). Guided Aspiration for Determining the Microbiological Aetiology of Aortic Vascular Graft and Endograft Infections. Eur. J. Vasc. Endovasc. Surg..

[38] Cernohorsky P., Reijnen M.M.P.J., Tielliu I.F.J., van Sterkenburg S.M.M., van den Dungen J.J.A.M., Zeebregts C.J. (2011). The relevance of aortic endograft prosthetic infection. J. Vasc. Surg..

[39] Shoai-Tehrani M., Pilmis B., Maury M.M., Robineau O., Disson O., Jouvion G., Coulpier G., Thouvenot P., Bracq-Dieye H., Valès G. (2019). Listeria monocytogenes-associated endovascular infections: A study of 71 consecutive cases. J. Infect..

[40] McGinigle K.L., Browder S.E., Strassle P.D., Shalhub S., Harris L.M., Minc S.D. (2021). Sex-related disparities in intervention rates and type of intervention in patients with aortic and peripheral arterial diseases in the National Inpatient Sample Database. J. Vasc. Surg..

[41] Mendes R.E., Sader H.S., Flamm R.K., Jones R.N. (2014). Activity of oritavancin tested against uncommonly isolated Gram-positive pathogens responsible for documented infections in hospitals worldwide. J. Antimicrob. Chemother..

[42] Jones R.N., Stilwell M.G. (2013). Comprehensive update of dalbavancin activity when tested against uncommonly isolated streptococci, *Corynebacterium* spp., *Listeria monocytogenes*, and *Micrococcus* spp. (1357 strains). Diagn. Microbiol. Infect. Dis..

[43] Sader H.S., Jones R.N., Stilwell M.G., Flamm R.K. (2014). Ceftaroline activity tested against uncommonly isolated Gram-positive pathogens: Report from the SENTRY Antimicrobial Surveillance Program (2008–2011). Int. J. Antimicrob. Agents.

[44] Sader H.S., Flamm R.K., Farrell D.J., Jones R.N. (2013). Daptomycin activity against uncommonly isolated streptococcal and other gram-positive species groups. Antimicrob. Agents Chemother..

[45] Lemaire S., Van Bambeke F., Appelbaum P.C., Tulkens P.M. (2009). Cellular pharmacokinetics and intracellular activity of torezolid (TR-700): Studies with human macrophage (THP-1) and endothelial (HUVEC) cell lines. J. Antimicrob. Chemother..

[46] Iqbal K., Milioudi A., Wicha S.G. (2022). Pharmacokinetics and Pharmacodynamics of Tedizolid. Clin. Pharmacokinet..

[47] Huang C., Lu T.L., Yang Y. (2023). Mortality risk factors related to listeriosis—A meta-analysis. J. Infect. Public Health.

[48] Samson R.H. (2004). Hypertension and the vascular patient. Vasc. Endovascular. Surg..

[49] Charlier C., Perrodeau É., Leclercq A., Cazenave B., Pilmis B., Henry B., Lopes A., Maury M.M., Moura A., Goffinet F. (2017). Correction: Clinical features and prognostic factors of listeriosis: The MONALISA national prospective cohort study. Lancet Infect. Dis..

[50] Legg J.S., Legg L.M. (2016). Abdominal Aortic Aneurysms. Radiol. Technol..

[51] Lyons O.T., Baguneid M., Barwick T.D., Bell R.E., Foster N., HomerVanniasinkam S., Hopkins S., Hussain A., Katsanos K., Modarai B. (2016). Diagnosis of aortic graft infection: A case definition by the management of aortic graft infection collaboration (MAGIC). Eur. J. Vasc. Endovasc. Surg..

[52] Lauri C., Campagna G., Aloisi F., Posa A., Iezzi R., Sirignano P., Taurino M., Signore A. (2023). How to combine CTA, ^99m^Tc-WBC SPECT/CT, and [^18^F]FDG PET/CT in patients with suspected abdominal vascular endograft infections?. Eur. J. Nucl. Med. Mol. Imaging.

[53] Mitjà O., Pigrau C., Ruiz I., Vidal X., Almirante B., Planes A.M., Molina I., Rodríguez D., Pahissa A. (2009). Predictors of mortality and impact of aminoglycosides on outcome in listeriosis in a retrospective cohort study. J. Antimicrob. Chemother..

[54] Lafon-Desmurs B., Gachet B., Hennart B., Valentin B., Roosen G., Degrendel M., Loiez C., Beltrand E., D’Elia P., Migaud H. (2024). Dalbavancin as suppressive therapy for implant-related infections: A case series with therapeutic drug monitoring and review of the literature. Eur. J. Clin. Microbiol. Infect. Dis..

[55] Lupia T., De Benedetto I., Bosio R., Shbaklo N., De Rosa F.G., Corcione S. (2023). Role of Oritavancin in the Treatment of Infective Endocarditis, Catheter- or Device-Related Infections, Bloodstream Infections, and Bone and Prosthetic Joint Infections in Humans: Narrative Review and Possible Developments. Life.

[56] D’Introno A., Cavallo M., Loparco F., Quarato L., Perrone L., Rollo V., Asabella A.N., Anglani A., Pennetta C.A., Ciracì E. (2024). Long-Time Conservative Treatment and Off-Label Use of Delafloxacin in Abdominal Aortic Graft Infection: Case Report. Ann. Case Rep..

[57] Nigo M., Luce A.M., Arias C.A. (2018). Long-term Use of Tedizolid as Suppressive Therapy for Recurrent Methicillin-Resistant Staphylococcus aureus Graft Infection. Clin. Infect. Dis..

